# Big Words, Halved Brains and Small Worlds: Complex Brain Networks of Figurative Language Comprehension

**DOI:** 10.1371/journal.pone.0019345

**Published:** 2011-04-27

**Authors:** Yossi Arzouan, Sorin Solomon, Miriam Faust, Abraham Goldstein

**Affiliations:** 1 Gonda Brain Research Center, Bar-Ilan University, Ramat Gan, Israel; 2 Racah Institute of Physics, Hebrew University of Jerusalem, Jerusalem, Israel; 3 Department of Psychology, Bar-Ilan University, Ramat Gan, Israel; University of Maribor, Slovenia

## Abstract

Language comprehension is a complex task that involves a wide network of brain regions. We used topological measures to qualify and quantify the functional connectivity of the networks used under various comprehension conditions. To that aim we developed a technique to represent functional networks based on EEG recordings, taking advantage of their excellent time resolution in order to capture the fast processes that occur during language comprehension. Networks were created by searching for a specific causal relation between areas, the negative feedback loop, which is ubiquitous in many systems. This method is a simple way to construct directed graphs using event-related activity, which can then be analyzed topologically. Brain activity was recorded while subjects read expressions of various types and indicated whether they found them meaningful. Slightly different functional networks were obtained for event-related activity evoked by each expression type. The differences reflect the special contribution of specific regions in each condition and the balance of hemispheric activity involved in comprehending different types of expressions and are consistent with the literature in the field. Our results indicate that representing event-related brain activity as a network using a simple temporal relation, such as the negative feedback loop, to indicate directional connectivity is a viable option for investigation which also derives new information about aspects not reflected in the classical methods for investigating brain activity.

## Introduction

Language comprehension is one of the most complex tasks handled by the brain. As such, it involves the coordinated activity of numerous regions each contributing a particular aspect of processing. Classically, “language regions” have been thought to be confined to the left hemisphere Wernicke's and Broca's areas, however recent evidence is accumulating indicating that other areas, especially in the right hemisphere, are necessary for various aspects of language. For example right-hemisphere activity is prominent during discourse processing [1], recognition of prosody [2], and comprehension of ambiguity [3] and novel metaphors [4]. Thus it is now obvious that language comprehension is not undertaken only by a couple of language “centers”, but it involves a large network of regions working in concert in a complex and dynamic manner [5]. Understanding such an intricate system calls for novel analytical methods.

A recent approach to the study of brain function portrays the brain as a network of anatomical or functional connections [6] and has the potential to be able to capture the complexity of brain function during mental operations. Complex networks can be analyzed and compared by representing networks abstractly as graphs (e.g., [7]–[8]), consisting of a set of vertices (nodes) linked by a set of edges (connections). Such graphs represent the topology of a network usually without taking into account the actual physical distance between vertices. Graph theory deals with the formal description and analysis of graphs and provides quantitative characterizations of any graph using a set of universal parameters. This approach has been successfully applied to the study of other complex systems such as metabolism [9], geophysical processes [10] and sociology [11].

Here we used topological measures to investigate how brain regions operate when understanding different types of expressions. The same brain and anatomical connections are used in comprehending textual material of different kinds. However, when reading expressions that require different processing styles, such as literal vs. novel metaphoric expressions, the various brain regions involved should contribute differently to the mechanisms of understanding. Thus the functional connectivity networks underlying different comprehension modes should reflect these changes in connectivity, even though they share the same anatomical connections. The aim was to utilize topological measures to qualify and quantify the functional connectivity of the networks used under various comprehension conditions.

To that aim we developed a technique to represent functional networks based on EEG recordings, taking advantage of their excellent time resolution in order to capture the fast processes that occur during language comprehension. We recorded brain activity while subjects read simple expressions and indicated whether they found them meaningful. Event-related potentials, which reflect activity time-locked to a particular event, in this case word presentation, were used in order to measure activity related only to the comprehension task. The networks were created based on the time-course of current source density estimates at 66 cortical areas derived from the event-related potentials.

Studies using graph theory analysis of functional brain networks have mainly used symmetrical measures of statistical association or functional connectivity between pairs of brain regions or sensors (e.g., [12]–[13]). Generally, these measures of connectivity are thresholded at some critical value to construct undirected graphs. Other studies have attempted to construct directed graphs using effective connectivity models based on causal influence estimates [14]–[15]. However the implementation of those models tends to be complicated and time consuming, and largely rely on prior theoretical knowledge. Here we opted for a third option and looked for a specific ‘physiological’ causal relation between areas, the negative feedback loop [16]. For each brain region pair, we tested whether activation of the first region was followed by activation of the second which in turn was followed by deactivation of the first region. Every pair that fulfilled the criterion was added as a link in the network. This method is a simple way to construct directed graphs using event-related activity, which can then be analyzed topologically. Negative feedback loops (NFLs) are of course not the only possible causal relation between brain regions, but they are ubiquitous in the nervous system. The presence of feedback loops was found in theoretical studies of oscillatory systems [17]–[18] that used differential equations to describe the dynamics of the system variables. Most of these models found NFLs to be an essential property of the system [19].

Our aim was to use this technique and apply it to EEG recordings of people involved in a language comprehension task [20] consisting of four types of simple expressions: word pairs with a literal semantic relation (‘burning fire’), conventional metaphors (“bright mind”), unfamiliar metaphors (“ripe dream”) and unrelated pairs (“indirect blanket”). These expression types have been shown to require different processing during comprehension [20]–[21]. A network was constructed for each expression type and was analyzed using various graph theory metrics. The aim of the research was twofold; the first aim was to test whether negative feedback loops can be used as a relatively simple measure of directed functional connectivity. The second aim was to show the usefulness of graph theory measures in the study of neuro-cognitive mechanisms. Our aim was not only to show that functional brain connectivity networks behave like complex scale-free small worlds, but also to derive new information about aspects not reflected in the classical methods for investigating brain activity. We hypothesized that slightly different functional networks will be obtained for event-related activity evoked by each expression type. The differences will reflect the special contribution of specific regions in each condition and the balance of hemispheric activity involved in comprehending different types of expressions.

## Method

### Ethics statement

All study procedures were approved by the committee of the Department of Psychology at Bar-Ilan University and all participants provided written informed consent.

### Participants

Twenty-eight right-handed native Hebrew speakers (12 men, 16 women, average age 23) participated in the experiment and received partial academic credit.

### Behavioral paradigm

Two-word expressions were presented in a random order, one word at a time, on a computer screen using white letters and black background. Stimuli on each trial were presented in the following time sequence: fixation cross (200 ms), first word (200 ms), blank (200 ms), and second word (200 ms). Participants were instructed to “judge whether the presented two-word expression conveys a meaning (be it literal or metaphoric) or does not convey a meaning as a pair”, and press a corresponding key. Response period was limited to 2 s and was followed by a 2 s inter-trial interval. A total of 96 Hebrew two-word expressions (24 per condition) were presented, consisting of conventional metaphoric (CM) expressions (e.g., *lucid mind*, *transparent intention*), novel metaphors (NM) drawn from poetry texts (e.g., *ripe dream*, *conscience storm*), literally related (LT) words (e.g., *burning fire*, *problem resolution*), and unrelated (UR) words (e.g., *indirect blanket, wisdom wash)*. The detailed experimental paradigm is reported in [4], [20].

### EEG Recording

EEG was recorded using a 65-channel geodesic net (Electrical Geodesics Inc.) with 250 Hz sampling rate, 0.1–100 Hz bandpass filter, and referenced to Cz. Impedances were kept below 50 kΩ. Data were further filtered (40 Hz) and referenced to an average reference offline. ERPs were time-locked to the onset of the second word of the pair. Epochs were 1000 ms long with a 100 ms pre-stimulus baseline. Trials with eye movement and other artifacts were removed.

### Data Preprocessing

ERPs were derived by averaging correctly classified trials on each condition for each participant, that is, LT, CM and NM pairs judged as conveying a meaning and UR pairs judged as not conveying a meaning. In order to estimate the neural sources of the waveforms for each condition, the average ERPs for each condition and subject were subjected to LORETA analysis [22]–[24]. LORETA calculates the three-dimensional current density distribution of the neural generators in the brain under the assumption that for each voxel the current density should be as close as possible to the average current density of the neighboring voxels (‘contiguity’). Computations were made using a three-shell spherical head model registered to the Talairach space of the brain's gray matter. The procedure yielded current density values for 2394 voxels, with a spatial resolution of 7 mm and a temporal resolution on the order of 4 ms. Voxels were labeled into 33 anatomical areas in each hemisphere according to the MNI305 atlas [25]. Data for each area were smoothed using a moving average of 10 samples (∼40 ms), then whitened with single value decomposition and normalized to the mean.

### Networks

Nodes were localized at the center of each anatomical brain region, and the activation of each node was calculated as the mean current density of all voxels in the region. Directional links between nodes were created by finding negative feedback loops with an algorithm adapted from [16]. For each pair of nodes, we tested whether during the time course of brain activity, activation of the first node was followed by activation of the second which in turn was followed by deactivation of the first region, regardless of the time length of activation or deactivation. A link was delineated between every two nodes (anatomical regions) that exhibited such a negative feedback loop anywhere along a discrete temporal dimension with time bins of 60 ms. Activation at a time-bin was defined as a current density value greater than the mean of that area, and deactivation was defined as a current density value lesser than the mean. Every node pair that fulfilled the criterion was added as a link in the network. The link was regarded as outgoing for the node that was activated first and incoming for the second node. A separate network was constructed for each condition.

### Network analyses

To analyze and quantify the connectivity of the emergent networks we used graph theory methods [26] and small-world networks analyses [27]. These were implemented using functions from the Complex Networks Analysis Package [28]. In the current study we calculated for each network the node degree distribution, betweenesss centrality, shortest path and clustering coefficient for the quantification of the small-world properties. These measures were calculated for each node, and averaged over all nodes to estimate the global characteristics of each network. The degree of a node is the number of connections that link it to the rest of the network, and the degree distribution refers to the degrees of all nodes in the network. Whereas random networks have a symmetrically-centered Gaussian degree distribution, complex networks generally have non-Gaussian degree distributions, often with a long tail towards high degrees. In scale-free networks the degree distribution follows a power law [29]. The clustering coefficient is calculated as the number of connections that exist between the nearest neighbors of a node as a proportion of the maximum number of possible connections. High clustering is taken to reflect high local efficiency of information transfer and robustness [30]. The shortest path measure is the minimum number of edges that must be traversed to go from one node to another. Short mean path-lengths reflect high global efficiency of parallel information transfer [31]. Betweeness is a measure of centrality indicating the relative importance of a vertex within the network. The betweeness of vertex *v* is the proportion of shortest paths between every two vertices in the network that pass through *v* (see [32] for a review).

## Results

### Network properties

**Figure 1:**
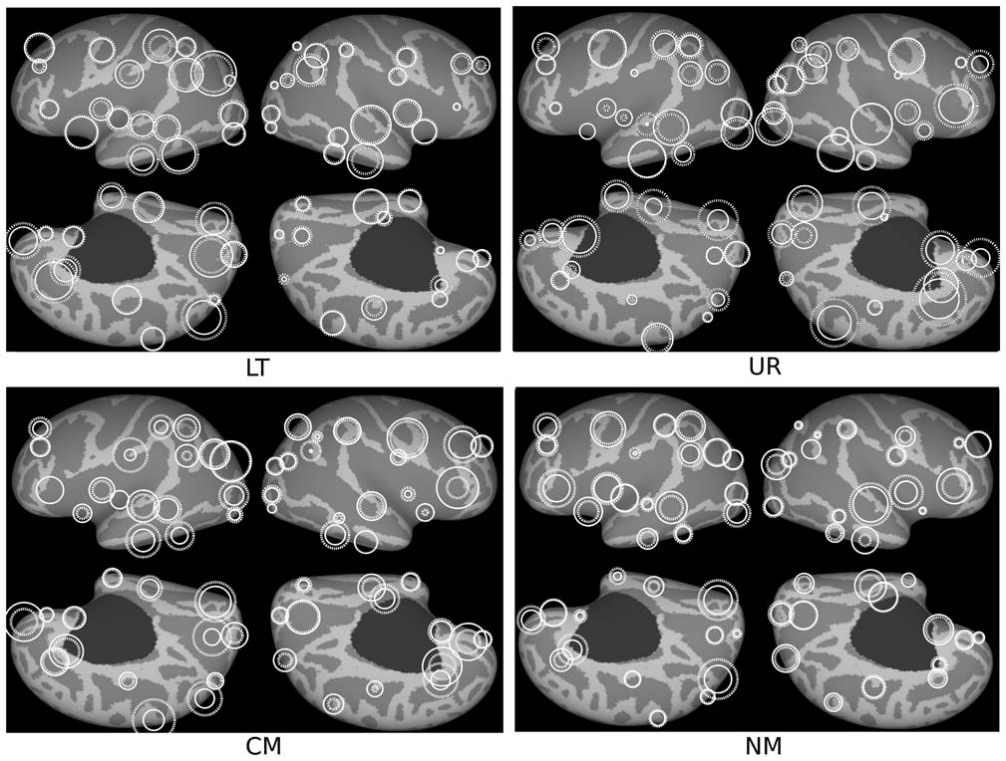
Graphical representation of the networks' main hubs for each expression type, located at the center each region. The diameter of the circles represent the node degree of the hub. Closed circles depict the outgoing degree, dotted circled depict the incoming degree.

This analyses process resulted in four directed networks with 66 nodes and 1687 directional links for the UR pairs, 1658 for the LT, 1603 for the CM and 1515 for the NM pairs, out of 4290 possible links in each network (Figure 1). The degree-distribution exponents (γ in the probability of degree k formula, p(k) = k^−γ^) ranged from 2.8 to 3.5, with a gradual decay reflecting the scale-free character of the networks, both for the incoming and outgoing links.

Average shortest path was relatively low (mean λ = 2.5) reflecting high global efficiency of parallel information transfer. However, the clustering coefficient (local efficiency) was also low (mean γ = 0.34), and not larger than an equivalent random net. This might be due to the low resolution of source localization in EEG and averaging of whole areas, which mask the activity at the local-network level.

### Hemispheres

A count of the inward and outward links for the nodes in each hemisphere indicated an overall balanced network hemispherically (Table 1). However, in the LT condition the number of links within the LH was significantly higher than the number of links within the RH, and this was reversed in the UR condition, χ^2^(3) = 57.4, p<0.0001). In both CM and NM conditions the leftward bias was not significant. A similar picture was obtained when examining the outgoing and incoming links. In the LT condition there were more outgoing links from the LH than from the RH, and more incoming links to the LH than to the RH. In contrast, for the UR condition there were more incoming and outgoing links in the RH than in the LH. This was statistically significant for both the incoming, χ^2^ (3) = 29.0, p<0.001, and outgoing, χ^2^ (3) = 32.2, p<0.001, links. Here again, both CM and NM conditions were more balanced, with a slight bias to the left which was not significant.

**Table 1:** Number of links in the networks of each condition.

	LT	UR	CM	NM
**Within hemisphere**				
RH	250	373	311	286
LH	479	316	377	384
RH/LH ratio	**0.52**	**1.18**	**0.82**	**0.74**
**Incoming to:**				
RH	609	746	689	652
LH	821	677	709	685
RH/LH ratio	**0.74**	**1.10**	**0.97**	**0.95**
**Outgoing from:**				
RH	592	734	643	587
LH	838	689	755	750
RH/LH ratio	**0.70**	**1.06**	**0.85**	**0.78**

**Figure 2:**
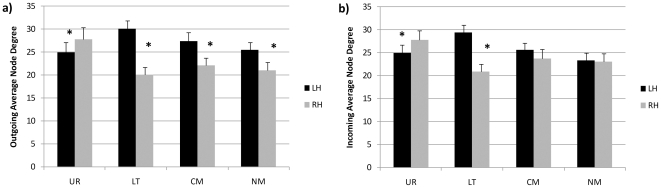
Average node degree for each condition and hemisphere. Left panel (a) shows the outgoing node degree. Right panel (b) shows the incoming node degree. Black bars: left hemisphere, grey bars: right hemisphere.

The average node degree between hemispheres and conditions was examined using analysis of variance. For the outgoing links the mean node degree was higher for the LH than for the RH (27 vs. 22.7), F(1,252) = 10.20, p<0.01. There was also an interaction between hemisphere and condition: the LH advantage was found in LT, CM, NM conditions but was reversed for the UR condition F(3,252) = 3.86, p<0.01. The interaction was significant, F(3,252) = 3.8,p<0.05, also for the incoming links, but no LH advantage was found for the metaphor conditions (Figure 2).

### Hubs

Hubs were defined as nodes with degrees higher than 2% of the total links in the network. The most connected hubs were different for the four conditions (Table 2). The measures for betweeness centrality paralleled those of the node degree. Thus, in these networks, nodes with the highest degree were also the central ones.

**Table 2:** Most connected nodes (hubs) for each condition in descending order according to their node degree.

LT	UR	CM	NM
L Angular (1.2)	R Medial Frontal (0.7)	L Sup Occipital (1.0)	R Sup Temporal (0.8)
L Medial Frontal (0.8)	R Sup Temporal (1.0)	R Precentral (1.2)	L Lingual (0.7)
L Sup Parietal Lob (0.7)	R Paracentral Lob (0.7)	L Lingual (0.7)	L Precuneus (0.7)
R Sup Temporal (1.2)	R Inf Frontal (0.8)	L Orbital (1.5)	L Precentral (1.2)
L Post Cingulate (1.2)	L Inf Temporal (1.0)	R Ant Cingulate (1.5)	L Inf Frontal (0.7)
L Fusiform (0.8)	L Precentral (1.0)	R Post Cingulate (1.0)	R Inf Frontal (1.4)
R Parahippocampal (1.0)	L Subcallosal (0.8)	R Inf Frontal (2.0)	R Sub-Gyral (1.0)
L Lingual (0.7)	R Fusiform (1.0)	R Middle Frontal (0.7)	L Insula (0.8)
L Orbital (0.7)	R Inf Occipital (1.1)	L Paracentral Lob (0.5)	L Middle Temporal (1.3)
L Supramarginal (1.1)	R Precentral (1.1)	R Rectal (1.6)	R Subcallosal (1.2)
R Inf Temporal (1.4)	R Parahippocampal (0.7)	L Ant Cingulate (1.3)	R Post Cingulate (1.0)
R Supramarginal(0.8)	R Subcallosal (0.7)	L Angular (1.1)	L Inf Parietal Lob (1.2)
	L Middle Temporal (1.4)	L Inf Temporal (0.6)	R Insula (0.6)
	R Lingual (1.3)		L Angular (1.0)
	L Middle Occipital (0.9)		R Parahippocampal (1.5)
	L Lingual (0.5)		L Sub-Gyral (0.9)
	R Angular (0.8)		L Rectal (0.9)

The in/out link ratio for each node is listed in parentheses.

In the NM condition the most connected node was the right superior temporal gyrus (rSTG), with more outward than inward links (in/out ratio  = 0.8). The right medial frontal gyrus was the main hub in the UR condition, also with an outward bias (0.7). The major hub in the CM condition was the left superior occipital gyrus (in/out ratio  = 1.0), and in the LT condition the left angular gyrus was the most connected node with a significant inward bias (1.2).

The only node that was a hub (>2%) in all conditions was the left lingual gyrus which had more outward than inward links (in/out  = 0.6). Two other nodes, the rSTG and the right parahippocampal gyrus, were hubs for NM, LT, and UR but not for CM, although their main direction differed between conditions. The rSTG had an outward bias for NM, was balanced for UR, and had an inward bias in LT. When counting only inward links, it was a major node for the CM condition as well. In contrast, the right parahippocampal gyrus had an inward bias for NM, an outward bias for UR, but was balanced for LT.

The left angular gyrus was a hub in all but UR conditions. Its direction was more in than out in CM and LT, but was balanced in NM. The right inferior frontal gyrus was a hub in all conditions but LT. It was mostly inward biased for CM and NM, but outwards biased for UR. Interestingly, the right inferior frontal gyrus had a very low node degree in the LT condition. Apart from the above nodes three additional nodes were found to be very connected in both the NM and UR conditions: left middle temporal gyrus, right subcallosal gyrus and left precentral gyrus. All had an inward bias, apart from the right subcallosal gyrus which had an outward bias for the UR condition. The right posterior cingulate was common to both metaphor types and had no directional bias. The precentral and rectal gyri were very connected also in both metaphor networks, but in different hemispheres: in the right hemisphere for CM and in the left for NM.

Hubs specific to the NM network included right and left insula, left precuneus and left inferior frontal gyrus (all with an outward bias), left inferior parietal lobule (inward bias), as well as bilateral temporal sub-gyri (balanced in direction). The CM network hubs comprised bilateral anterior cingulate (in), right middle frontal gyrus and left paracentral lobule (both out). The most connected nodes when comprehending literal expressions were mainly regions involved in reading words, such as the left fusiform, lingual and angular gyri as well as bilateral supramarginal gyri.

## Discussion

Our results indicate that representing event-related brain activity as a network is a viable option for investigation. In particular, the use of a simple temporal relation such as the negative feedback loop to indicate directional connectivity resulted in networks consistent with the literature in the field. Several of the brain regions found to be the most central and functionally connected are areas that have previously been reported as active during language tasks in imaging studies. However, the networks parameters revealed information not available through regular analyses. Some of the most connected regions in our networks are not frequently reported as having increased activation in comprehension tasks. Thus, the directional connectivity yields important information that is not reflected in general activity measures.

The hemispheric balance indices of the network underlying literal expressions show a left hemispheric bias, in accordance with the literature. In contrast, the UR network was right biased, probably reflecting the use of coarse coded concepts when searching for meaning [33]. Both types of metaphors, conventional and novel, were handled by more balanced networks, indicating that information flow from the two hemispheres is needed to understand them. When comprehending meaningful expressions, the LH had a higher average node degree, indicating that the regions in that hemisphere had more connections, both for incoming and outgoing links. The reverse was true for UR in which the RH had greater connectivity overall. In a previous study [4] we showed that both hemispheres are activated when comprehending figural meanings. The present findings demonstrate that both hemispheres contribute and share information, although the specific information flow differs depending on the type comprehension needed.

The differences between the functional networks are better described by considering the main hubs or central nodes which were common for various expression types and those which appeared only in particular conditions. The only node which was found as a main hub in all conditions was the left lingual gyrus. The left lingual gyrus is frequently reported to be active when recognizing words and has been shown to be more active for words than pseudowords [34]. Thus, it should naturally appear as a hub in each of the four networks. Its direction, mostly outward connections, is consistent with its role of feeding the information about the word to other areas.

A second common hub was the right superior temporal gyrus. The superior temporal gyri have been suggested to have a role in semantic integration, with an advantage of the right hemisphere for integrating multiple distantly related concepts and activating large semantic fields [33]. The coarse coding of the right hemisphere is useful in complex language tasks that require integrating contextual information or bridging between seemingly unrelated words as in the present experiment. Our results indicate, however, that the flow of information in the integration mechanism is different for the various expression types. In the NM network the directionality of rSTG had a slight outward bias, but had an inward bias for conventional metaphors and literal expressions. This suggests that semantic integration is not merely a passive process but an active one involving both transmitting as well as receiving information from various sources.

An additional common hub was the right parahippocampal gyrus, which is frequently found in tasks involving contextual memory [35] as well as social context such as when understanding sarcasm [36]. When searching for the meaning of seemingly unrelated words contextual information has a key role. Interestingly, most connections in that node were inbound in the NM network, but outbound in the UR network. It can be speculated that when the meaning of novel metaphoric expressions was successfully reached this was partly based on integrating such contextual information. In contrast, with unrelated word pairs, contextual information was used as a search strategy which turned to be unsuccessful. For conventional or ‘dead’ metaphors for which the figurative meaning is the salient one, using contextual information would be detrimental thus the parahippocampal gyrus did not emerge as a hub.

The left angular gyrus was a main hub in the networks for all meaningful expressions. It is part of the word reading network and is functionally connected with visual association areas in the occipital and temporal lobes as well as language areas [37]. It is presumed to be involved in mapping visually presented inputs onto linguistic representations [38]. Interestingly, patients with damage to the left angular gyrus are unable to comprehend the metaphorical meaning of common proverbs [39].

Comprehension of novel metaphors was implemented in a functional network in which the most connected areas included the left precuneus, left inferior frontal gyrus, as well as left inferior parietal lobule and left angular gyrus. The precuneus is thought to act as an associative region involved in metal imagery and has wide connections with the inferior parietal lobule and angular gyri [40]. Together with the inferior frontal gyrus it is active in episodic memory tasks [41], and has been considered as a parietal prefrontal hub [32]. Its centrality in novel metaphor comprehension is consistent with its associative and working memory roles. Comprehending novel metaphors entails retrieving information about distant concepts which are then imagined in order to find the parallels between the source and the target. The right and left insulae also turned up as very central areas for novel metaphors. Previous studies using this type of stimuli have also found increased insular activation [42]–[43].

In contrast, the network for conventional metaphors included the left superior occipital gyrus, the anterior cingulate, the right middle frontal gyrus and the left paracentral lobule. The left angular, lingual gyrus, and right posterior cingulate, as well as more frontal regions (precentral gyrus, inferior frontal, and rectal gyri) were common to both types of metaphoric expressions. However, the laterality of the precental gyrus and the inferior frontal gyrus was opposite for the two conditions, right hemisphere for conventional metaphors and left hemisphere for novel ones.

In sum, the characteristics of the networks produced using the NFL method with ERPs have the potential to reveal important information about the cognitive mechanisms they represent. Of course NFLs are not the only type of functional connection between areas, but they are ubiquitous in the nervous system, and as shown here, they yield a coherent representation of the activity of the brain under various task conditions. It is important to note, however, that networks derived from EEG data were not completely compatible with the expected small-world connectivity in the cortex. This is probably due to the lack of spatial resolution of the LORETA solutions and the use of whole regions as nodes. Such low scale data can only reveal long-range connections between areas and is blind to within-area local connections. It is possible that brain recordings with better spatial resolution such as MEG will provide the necessary information to render small-world networks using this method.
